# The Effect of Laser Remelting during SLM on Microstructure and Mechanical Properties of CoCrFeNiNb_0.25_

**DOI:** 10.3390/ma17092061

**Published:** 2024-04-27

**Authors:** Zhiyuan Yang, Chan Guo, Tao Sun, Jinpeng Hu, Xiaomei Feng, Yifu Shen

**Affiliations:** College of Materials Science and Technology, Nanjing University of Aeronautics and Astronautics (NUAA), Nanjing 210016, China; yzygkd@nuaa.edu.cn (Z.Y.); guochan@nuaa.edu.cn (C.G.); suntao_nuaa@outlook.com (T.S.); jphu_wsf@nuaa.edu.cn (J.H.)

**Keywords:** SLM, HEA, laser remelting, microstructure, mechanical properties

## Abstract

A sub-eutectic high-entropy alloy composed of CoCrFeNiNb_0.25_ was prepared using a combination of mechanical powder mixing and selective laser melting (SLM). The mechanical properties of the alloy were enhanced by employing an interlayer laser remelting process. This study demonstrates the feasibility of using mechanical mixing and SLM to form an CoCrFeNiNb_0.25_ alloy. The interlayer laser remelting process can effectively promote the melting of Nb particles introduced by mechanical mixing, release the stresses near the unfused Nb particles, and reduce their degradation of the specimen properties. The results indicate that the CoCrFeNiNb_0.25_ alloy, prepared using the interlayer laser remelting process, had an average microhardness of 376 HV, a tensile strength of 974 MPa, and an elongation at break of 10.51%. This process offers a viable approach for rapidly adjusting the composition of high-entropy alloys for SLM forming.

## 1. Introduction

The exploration of metals has often involved adding small amounts of other elements to a major element to improve its properties in specific aspects. This practice has led to the design of alloy compositions based on the traditional binary phase diagram. However, in order to explore new alloy systems, Yeh [1,2] proposed the concept of equimolar poly-alloy systems, also known as high-entropy alloys, in 2004. High-entropy alloys have a composition that is more complex than traditional alloys, with a ratio of components that is close to equimolar. As a result, they exhibit many effects that differ from those of traditional alloys. These effects include the high-entropy effect, hysteresis diffusion effect, lattice distortion effect, and cocktail effect [3,4,5].

Most high-entropy alloys are formed by casting after conventional vacuum arc melting or induction melting [6,7], or by mechanical alloying followed by spark plasma sintering [8,9]. However, during the melting process, the casting alloys are also subjected to several remelts to eliminate inhomogeneities in the alloy composition. In addition to the fact that both processes are very time-consuming, some high-entropy alloys need to be formed with a high cooling rate to inhibit the precipitation of intermetallic compounds in the solid solution phase. In summary, the traditional process of forming high-entropy alloys has a longer cycle time, higher cost, and even fewer advantages when corresponding to complex geometries. Therefore, the rapid solidification forming of high-entropy alloys has become a new technology widely used in research and production.

For instance, Brif et al. [10] investigated the possibility of forming high-entropy alloys through selective laser melting technology using pre-alloyed powders prepared by the aerosolization method. They successfully produced CoCrFeNi high-entropy alloys with equimolar ratios, which exhibited both high strength and ductility, by varying the layer thicknesses and annealing processes.

The microstructure and macroscopic properties of high entropy alloys are often a composite of the characteristics of the elements in their components. The types and contents of the elements used affect these properties. The introduction of new elements into an already mature system can cause the re-alloying of high-entropy alloys, resulting in specific improvements in performance. Yu et al. [11] successfully prepared AlCoCrFeNi_2.1_ eutectic high-entropy alloys by adding Al elements to the CoCrFeNi high-entropy alloys mentioned above. Li [12] also conducted research in this area. The nearly single-face-centered cubic phase CoCrFeMnNi high-entropy alloy was successfully prepared using the selective laser melting process. The pre-alloyed powder was used with a laser energy density parameter of 74 J/mm^3^ for the alloy. The relative density of the alloy was 98.2%. However, the use of pre-alloyed powders is expensive and time-consuming, making it unsuitable for small-scale engineering applications. Chen [13] and colleagues prepared CoCrFeMnNi high-entropy alloys using CoCrFeNi pre-alloyed powders and Mn elemental powders via selective laser melting. The mechanical properties of the resulting specimens were similar to those reported previously, but with lower elongation. Optimization was necessary to eliminate inherent defects caused by mechanical powder mixing during the process.

In summary, there are few publications on the preparation of CoCrFeNiNb_0.25_ alloys using a combination of mechanical powder mixing and selective laser melting processes. The mechanism of defects caused by mechanical powder mixing is not yet clear. The aim of this paper is to examine the impact of interlayer laser remelting on the microstructural evolution and mechanical properties of specimens created through the combination of mechanical powder mixing and selective laser melting processes.

## 2. Materials and Methods

### 2.1. Powder Preparation

**Figure 1:**
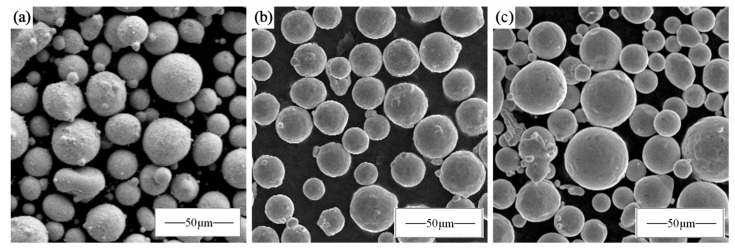
Powder morphology: (**a**) CoCrFeNi; (**b**) Nb; (**c**) CoCrFeNiNb_0.25._

The CoCrFeNi pre-alloyed powder and Nb monolithic powder used in this experiment were aerosolized and prepared as spherical powders with a particle size distribution of 15–53 μm. Then, 5.8 at% Nb monolithic powder was mixed with the CoCrFeNi pre-alloyed powder in a planetary ball mill for 1 h at a ball-to-material ratio of 5:1 and a rotational speed of 180 rpm, respectively. The microscopic morphology is shown in Figure 1, where (a) is CoCrFeNi pre-alloyed powder, (b) is Nb monolithic powder, and (c) is mechanically mixed powder. The powder had high sphericity and good flowability, which met the requirements of metal printer printing.

### 2.2. SLM Process

**Figure 2:**
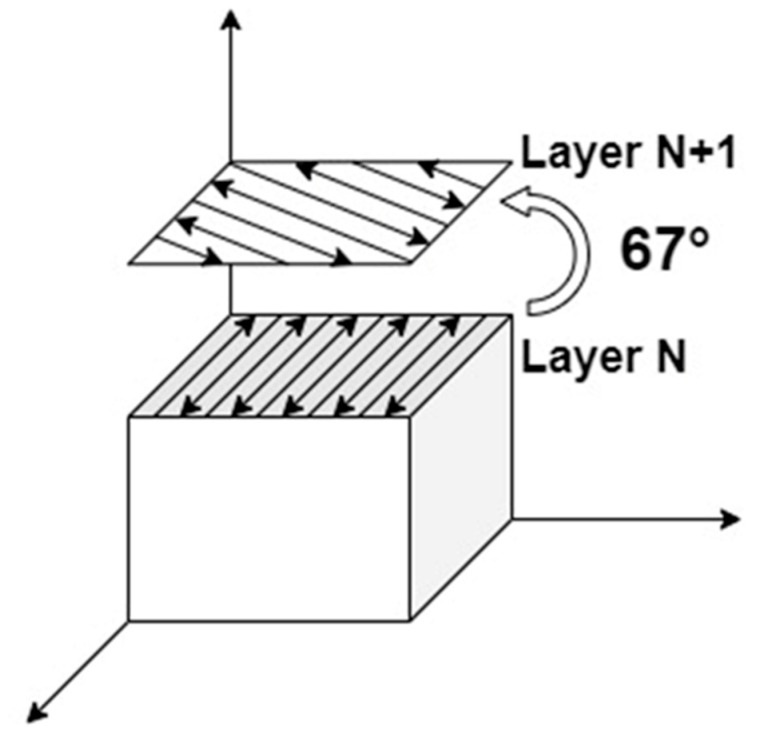
SLM strategy.

SLM forming was performed by an SLM machine equipped Nd: YAG laser with a maximum power of 500 W and a spot diameter of 110 μm. Then, 316 L stainless steel was used as the substrate for the SLM forming process, the temperature of the powder supply cylinder was preheated to 80 °C, the substrate was preheated to 150 °C to minimize the residual thermal stresses [14], and argon was used to displace the air in the forming compartment to keep the oxygen content below 400 ppm. Through preliminary experiments, the optimized forming parameters for the alloy powder were 200 W laser power, 600 mm/s scanning speed, and 40 μm layer thickness, which could be avoided by using a smaller layer thickness to avoid the warping of the sample caused by the concentration of thermal stresses [15], but, due to the limitations of the selective laser melting and laying process, the layer thickness should be commensurate with the average distribution of powder particle size. A smaller layer thickness would result in a smaller alloy powder, which would not be conducive to cost reduction in practical production. Therefore, in this paper, laser remelting was used to achieve the effect of thermal stress reduction. During sample forming using the laser remelting strategy, the remelting step can result in a shallower layer during forming due to the higher laser reflectivity of the solidified surfaces and the higher thermal conductivity of the bulk than the powder. At the same time, the layer-by-layer scanning characteristic of the selected zone laser melting technology can evenly distribute the elemental segregation caused by remelting in the sample to ensure the uniformity of the mechanical properties of the parts. The number of interlayer laser remelting scans was selected as 2 times, and the SLM scanning strategy is shown in Figure 2. The scanning direction was deflected by 67° between the N-layer and the N+1-layer to ensure that the formed part eliminated the anisotropy in the properties [16]. 

**Figure 3:**
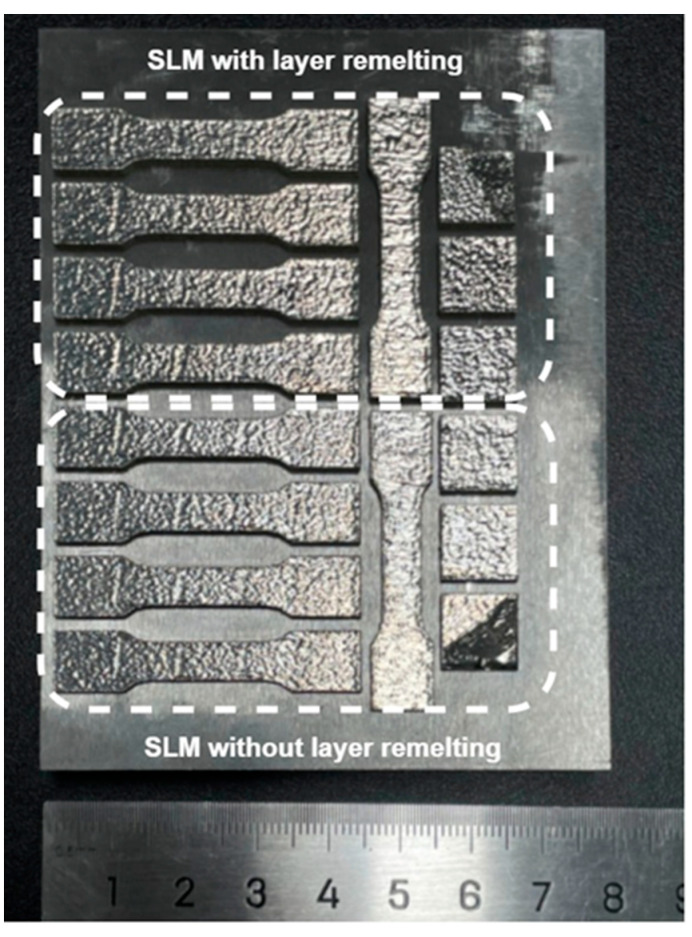
Macro morphology of the formed sample.

The final formed sample is depicted in Figure 3. It exhibited satisfactory surface quality, with no evidence of dimensional distortion.

### 2.3. Microscopic Characterization

After the forming of the SLM process, the samples were cut from the substrate by wire cutting, cleaned with ethanol and ultrasonic waves, and dried in a vacuum drying oven. Three densitometric measurements were performed and averaged for each sample using Archimedes’ principle, respectively.

To analyze the microstructure of the samples, 450 mesh, 800 mesh, 1000 mesh, and 2000 mesh sandpapers were used to polish the samples in turn. Then, the samples were polished by an LMP-3S type metallographic polishing machine and diamond polishing spray. Etching was carried out using diluted aqua regia. Observation was conducted using a Zeiss Axio Vert microscope (Zeiss, Oberkochen, Germany). XRD tests were carried out using an Empyrean intelligent X-ray diffractometer (Malvern Panalytical, Almelo, The Netherlands). The device was equipped with a metal ceramic X-ray tube (Cu target) with a maximum power of 2.2 kW, and the parameters taken for the test program in the experiments were a test angle of 20° to 90°, a step size of 0.026°, and a scanning speed of 8°/min.

The microstructure was observed using a LYRA3 GMU type aggregated ion beam scanning electron double beam electron microscope from TESCAN (Brno, Czech Republic). Phase composition was observed using EBSD.

### 2.4. Mechanical Properties

The microhardness of the samples was measured on an MH-500 micro Vickers hardness tester (Hengyi, Shanghai, China). The Vickers hardness tester measurements were as follows: The test load was set to 1000 gf and the hold time was set to 15 s. The measurement plane was perpendicular to the building direction, and each sample was measured along a straight line with a spacing of 200 μm. The spacing between adjacent measurement points was 200 μm. The average value of 15 measurement points was taken. The room temperature tensile test was performed on a WDW-100 H universal testing machine (Zhongluchang, Jinan, China) with a loading speed of 1 mm/min.

## 3. Results and Discussion

### 3.1. Densification

**Figure 4:**
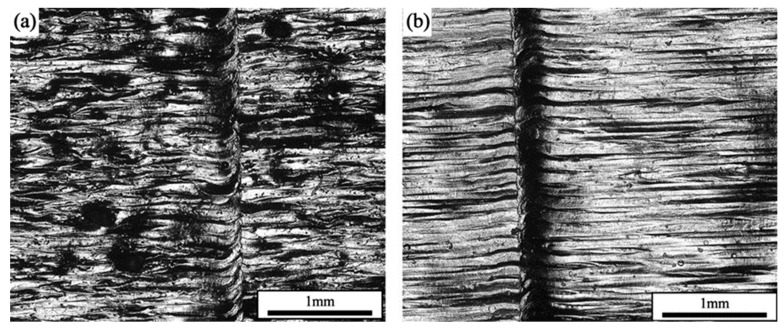
Surface morphology of formed samples: (**a**) SLM; (**b**) SLM + LR.

Figure 4 shows the samples formed with different scanning strategies. During the first scanning of the forming plane by the laser, the unmelted area of powder particles blocked the full flow of the melt pool, which reduced the quality of the surface, and also introduced particles that were not metallurgically bonded to the surface of the part. It was seen that, at the same laser power and scanning speed, the surface roughness of the parts prepared with the single scanning strategy was better than that with the laser remelting strategy. The formation of pores further reduced the densification of the formed part. Using the laser remelting process, a second scan was performed on top of the first laser scan to effectively preheat the part. The resultant melt pool had a higher fluidity and a longer residence time, which helped to form a smoother surface, and the powder spreading tape was less perturbed during layer-by-layer buildup, resulting in a more uniform spreading of the powder. At the same time, the remelting process achieved smaller and shallower melt pools with multiple scans of the current forming layer. On the one hand, because the thermal conductivity of the powder and the surface of the preliminary formed sample were different, the heat of the powder irradiated by the laser was easy to accumulate, and the surface of the formed sample could better transfer the heat input. On the other hand, because of the difference in the degree of flatness of the surface of the powder and the surface of the preliminary formed sample, the surface of the powder was rough, and it absorbed the laser energy to a greater extent, and the surface of the formed sample could output a part of the laser energy to the outside world in the form of a reflection. The combined effect of the two could form a shallower melt pool on the surface of the formed layer, which to some extent would be equivalent to a reduction in the layer thickness parameter. However, the overall heat input of the remelting scanning process was, therefore, smaller than that of the single scanning process and would require further fine-tuning of the laser power.

In practical engineering applications, the purpose of improving the bonding quality between layers is often achieved by reducing the layer thickness, but the layer thickness setting depends on the particle size distribution of the alloy powder. If the layer thickness is too small, the particle size is larger than the layer thickness of the powder and cannot be attached to the forming layer and the powder spreading tape is scraped off. To avoid this, according to the adjustment of the layer thickness of the raw materials, a smaller particle size distribution of powder needs to be selected. At this stage, the alloy powder with good sphericity and fluidity is usually prepared by aerosolization, and its process principle determines the diminishing marginal benefit. The finer the powder often means that the cost increases exponentially; therefore, a remelting process with multiple scans is more valuable in practical engineering applications.

**Figure 5:**
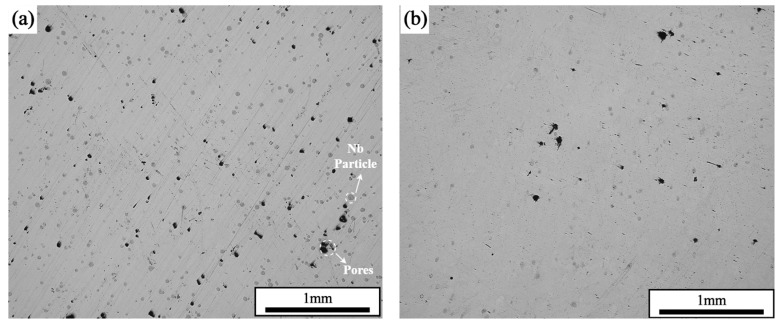
Cross-sectional morphology: (**a**) SLM; (**b**) SLM + LR.

Under the parameters of a laser power of 200 W and a scanning speed of 600 mm/s, the densities of the samples prepared by single scanning were 99.33%, and the densities of the samples prepared by two interlayer laser remelting scans were 99.96%. The cross-sectional morphology of the formed samples is shown in Figure 5, which shows that the samples that adopted the SLM + LR process had fewer pores, which was the main reason for the increase in densities. It was observed that the sample with the SLM + LR process had less porosity, which was the main reason for the increase in density, and the second phase unfused Nb particles interspersed within it were significantly reduced.

### 3.2. XRD

**Figure 6:**
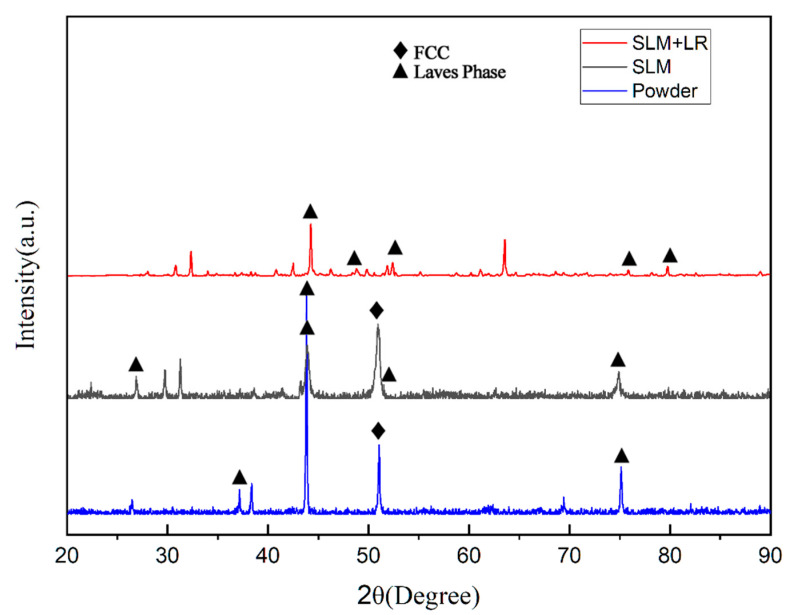
XRD diffraction peaks.

Figure 6 shows the XRD diffraction peaks of the raw material powder prepared by the simple mechanical mixing method and the two process forming samples. It was observed that the diffraction peaks of the raw material powder prepared by the mechanical mixing method were only a simple superposition of the CoCrFeNi pre-alloy and the Nb monomers, suggesting that it did not undergo the mechanical alloying in the ball milling process. In specimens formed using SLM, the diffraction peaks of the Laves phase with a hexagonal structure began to appear on the side of the FCC (111) peak. This resulted in the FCC phase losing its growth selective orientation, and the (111) peak decreased. In contrast, the XRD diffraction peaks of the specimens prepared by selective laser melting (SLM) broadened significantly and shifted to the left as a whole, in comparison with the specimens prepared by the traditional casting method [17]. Furthermore, the analysis of the Bragg’s formula revealed that when the wavelength of the X-rays was maintained, the diffraction angle θ diminished, resulting in an expansion of the crystal plane spacing d. This phenomenon can be reasonably interpreted as an indication of a considerable number of lattice aberrations in the specimens. Moreover, the equations derived by Woo and Jiang et al. [18] based on the Williamson–Hall (WH) theory indicated that a significant amount of residual stresses existed within the specimen due to the nature of the zone-selective laser melting process itself. The half-height width of the diffraction peaks of the specimens shaped by the SLM + LR process was narrowed, the FCC (111) peak was further reduced, and the diffraction peaks did not shift. These observations indicated that the use of the LR process was able to reduce the residual stresses and lattice distortions in the specimens.

### 3.3. SEM 

**Figure 7:**
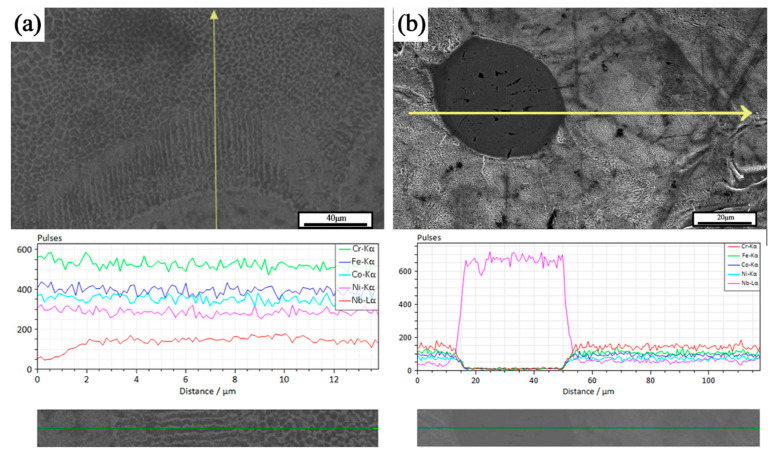
EDS line sweep: (**a**) segregation-driven phase transitions; (**b**) diffusional transformation.

Figure 7 shows the EDS line scan results of some areas in the formed sample. Two types of organizational structures were observed, the first being spherical second-phase particles, and the second being columnar crystals diffused outward with the FCC matrix as the center. According to the EDS results, the second phase particles were unfused Nb particles. These observations were consistent with the study of He et al. [17]. As shown in Figure 7, the dissociated eutectic, eutectic, and hypereutectic tissues were distributed in different degrees. Around the Nb particles, per-eutectic organization dominated by the bright white coarse Laves phase was observed. Away from the Nb particles, the dissociated eutectic organization dominated by the dark gray FCC matrix was presented. The lamellar eutectic organization was observed at the transition position between the two. The analysis suggests two different precipitation mechanisms for the Laves phase within the tissue, depending on whether the Nb particles were completely dissolved in the matrix. It is important to note that there were different degrees of unfused Nb particles in the sample tissue, and the investigation in this section refers specifically to the second-phase Nb particles that fused with the matrix to some extent.

When the Nb particles completely melted and formed a uniform CoCrFeNiNb_x_ liquid phase, the outward precipitation of the Nb-rich Laves phase, at the same time, due to the higher melting point of Nb, the liquid phase in this region absorbed more laser energy than the CoCrFeNi matrix, so the temperature was higher, and the surrounding organization from the cellular grains gradually led to laminar grain excess. Selected laser melting samples in the microstructure were mainly temperature gradient G and cooling rate R. Smaller G/R was conducive to the formation of cellular organization, in the high-temperature liquid phase and low-temperature liquid phase interface. The influence of the temperature gradient G dominated, generating the formation of laminar grains. With the increase in the distance from the interface, the temperature gradient G became smaller. When the selected laser melting process of rapid solidification characteristics of the obvious cooling rate R dominated, the influence of the temperature gradient G became smaller. The influence of the cooling rate R dominated, and thus a large number of cellular grains were generated. The existence of obvious Nb-poor regions in the results of the EDS line scans were also proof of this point.

When the Nb grains were not completely melted, a concentration gradient of Nb elements was generated between the second phase grains and the matrix, and the Laves phase was formed by diffusion from the second phase grains into the matrix. During the diffusion, the Nb element invaded the matrix and was exchanged with the elements in the matrix. Because the atomic radius of the Nb element was larger than that of Co, Cr, Fe, and Ni elements, a larger lattice distortion occurred in the region of the transition layer in the process of Nb diffusion, and therefore a larger microstress was also generated in the region. The SEM results showed that there were some tiny pores at the center of the unfused Nb particles because the atomic radius of Nb was different from that of Co, Cr, Fe, and Ni elements. Atomic radius and Co, Cr, Fe, and Ni matrix element atomic radius differences were large. The diffusion rate had a significant difference and, in the diffusion process, Nb atom diffusion flux and matrix atom entry flux were not equal, resulting in the Kirkendall effect. This can also partially explain the phenomenon that the densities of the formed samples failed to reach the theoretical full densification.

From a comprehensive point of view, the Laves phase precipitation behavior during the preparation of CoCrFeNiNb_0.25_ samples by mechanical mixing combined with selective laser melting can be classified into two types, one of which was a segregation-driven phase transition, i.e., the Nb elements solidly dissolved in the matrix precipitate to form the Laves phase. The other was the diffusion of Nb from incompletely melted Nb particles into the matrix, during which Nb-rich Laves phases dominated the diffusion process. Both precipitation behaviors can be enhanced by generating a diffuse Laves phase in the FCC matrix.

**Figure 8:**
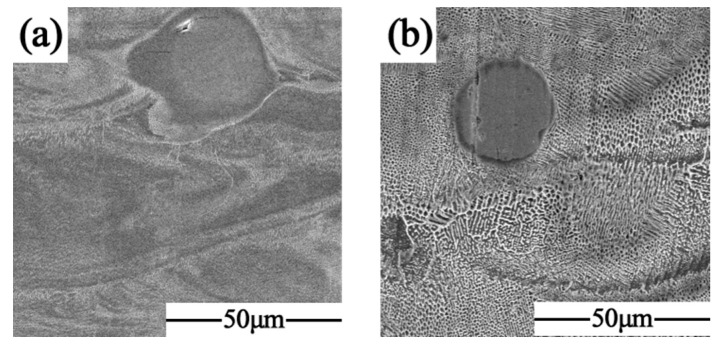
SEM diagram: (**a**) SLM + LR; (**b**) SLM.

Figure 8 shows the metallographic micrographs of the formed samples with different scanning times at the same volumetric energy density. It can be seen that the unfused Nb particles were gradually dissolved under the action of laser remelting to form a diffusion layer with the FCC substrate, and the Nb atoms also fully flowed in the remelting to produce a melt pool under the Marangoni effect to form the dispersed Laves phase in the solidified melt channel. The tissue morphology underwent a transformation from the original cellular crystals into a fine lamellar eutectic with varying orientations. Furthermore, the orientation of the lamellar eutectic organization was disrupted by the laser remelting process, resulting in a notable reduction in anisotropy.

### 3.4. EBSD

**Figure 9:**
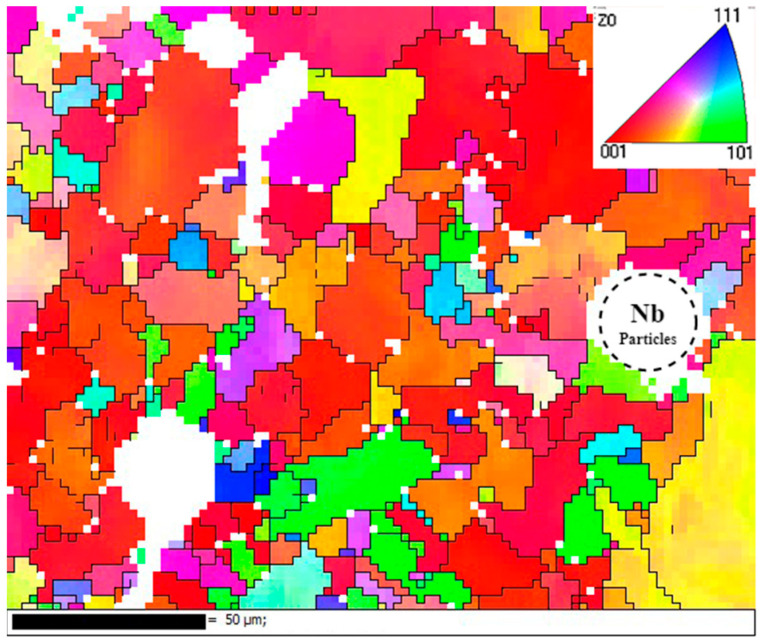
IPF diagram.

Figure 9 shows the grain orientation of the formed specimen. It can be seen that the fine grains were mainly concentrated in the vicinity of unfused Nb particles, and the main grain orientation was in the (001) direction. This was because the radius of the Nb atoms was much larger than that of the elements in the matrix, which produced a strong lattice distortion effect and thus induced grain refinement.

**Figure 10:**
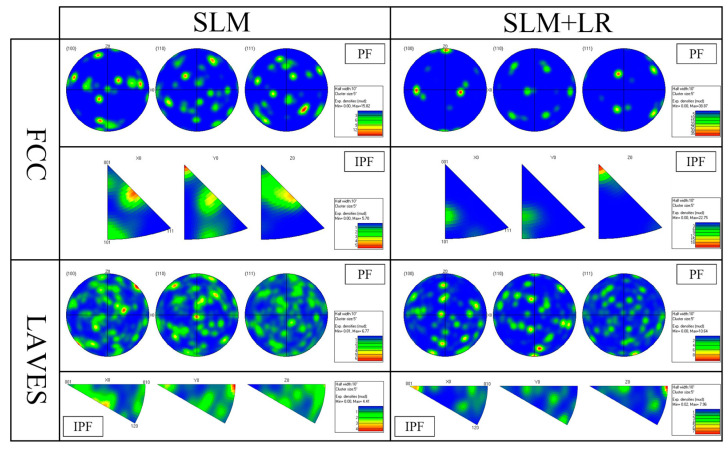
PF and IPF diagram.

Figure 10 shows the IPF plots of the samples formed by the SLM process versus the SLM + LR process. It can be seen that the SLM-formed specimens showed more weave in the {100} direction. This was mainly due to the fact that more Nb entered the matrix during the remelting process, causing the concentration of Nb elements in the local area to reach the eutectic point and, thus, eutectic reaction occurred.

**Figure 11:**
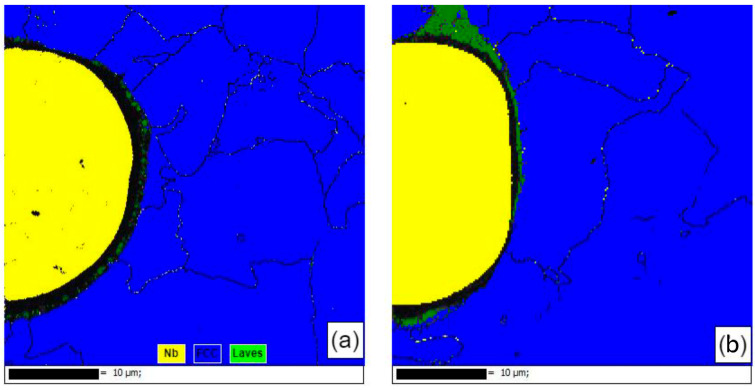
EBSD phase analysis diagram: (**a**) SLM; (**b**) SLM + LR.

Figure 11 shows the EBSD phase compositions near the second-phase particles of the samples formed using single scan forming and those formed using the remelting process. The second-phase Nb particles of the single-scan formed sample were well spherical and subcircular. They did not melt during the forming process but were wrapped by the molten liquid phase of the matrix to form inclusions. In this process, the second phase Nb particles were only affected by the thermal diffusion of Nb atoms into the matrix, and the zero-resolution points near the Nb particles were Laves phases that were not recognized by the database due to severe lattice distortions, while the zero-resolution points inside the Nb particles were pores created by the Kirkendall effect [19] in the thermal diffusion. This was used to analyze the residual stresses in the vicinity of the Nb particles. Comparing the samples formed by the remelting process, it was seen that the sphericity of the Nb particles decreased, the whole was ellipsoidal and was partially melted during the forming process, the interior of the Nb particles was dense, and the percentage of the surrounding zero-resolution points decreased. It was observed that there was no obvious lattice aberration in the transition region of the Nb particles to the substrate in the Laves phase, the diffusion layer became thicker, and the second phase of the Nb particles was more tightly bonded to the substrate. Meanwhile, according to the EBSD results, it was also seen that the stresses were mainly concentrated in the vicinity of the second-phase Nb particles, which was reflected by the zero-resolution points caused by lattice distortion near the second-phase Nb particles. It was analyzed that the stress in this region was mainly due to the inconsistency of the composition of the second-phase Nb particles and the matrix, which had a large difference in the coefficient of thermal expansion. The surface temperature of the sample was higher in the forming process, and the melted matrix was wrapped with the second-phase Nb particles. The shrinkage rate of the two was inconsistent with the solidification of the forming layer after cooling, and the diffusion of the elements occurred under the influence of the heat and the concentration gradient (a common effect of the solidification shrinkage and the Kirkendall effect). The solid phase interface between the two had a large stress residue under the joint action of the Kirkendall effect and solidification contraction. The stresses generated by the larger number of second-phase Nb particles were difficult to eliminate by optimizing the process parameters and therefore manifested as thermal cracking defects on a macroscopic scale. The above analysis shows that the remelting process can effectively promote the reduction of the second-phase Nb particles, promote the increase of their content in the matrix, and effectively release the stress near the second-phase Nb particles.

### 3.5. Mechanical Properties

**Figure 12:**
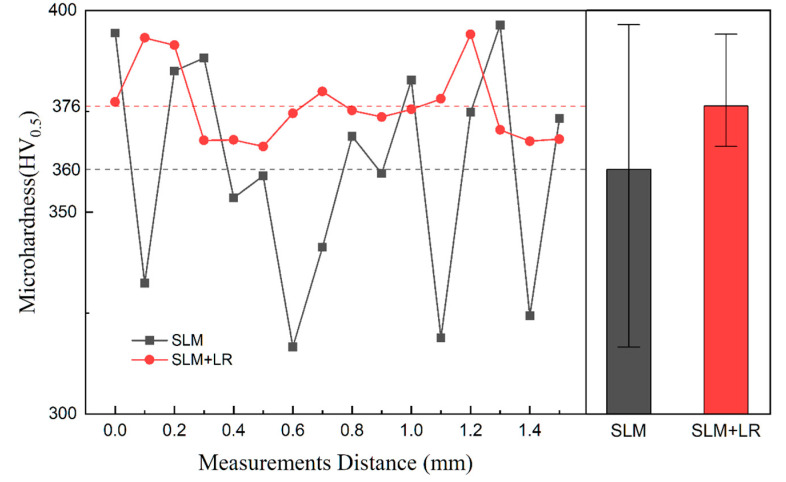
Microhardness distribution.

Figure 12 shows the microhardness distribution of CoCrFeNiNb_0.25_ alloy samples prepared by different processes, and it can be seen that the average hardness of the samples using the remelting process was increased by 16 HV, and the hardness uniformity was better. The increase in Nb elements in the matrix improved the microhardness of the formed samples, and the remelting process increased the dissolution degree of the second phase Nb particles, which in turn improved the Nb content in the matrix. At the same time, the increase in hardness uniformity was due to the remelting process effectively disrupting the grain morphology, resulting in a significant reduction in the anisotropy of the sample’s mechanical properties.

**Figure 13:**
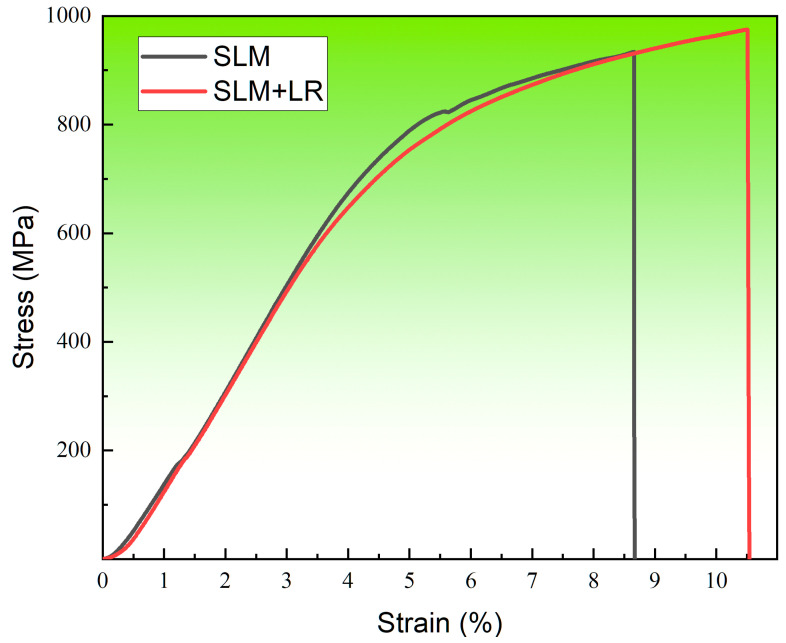
Tensile curve.

Figure 13 shows the room temperature tensile curve of the forming sample under the two processes. The selected laser melting process parameters were consistent, with a laser power of 200 W and a scanning speed of 600 mm/s. Single scan forming had a tensile strength of 934 MPa and an elongation of 8.66%. The laser remelting process had a tensile strength of 974 MPa and an elongation of 10.51%. It was seen that the alloy samples with the remelting process had higher strength than the single scanning samples, and the plasticity was also improved. On the one hand, it was because the remelting process reduced the structural defects in the forming samples so that the matrix densification rose, and the combination was more compact. On the other hand, the remelting process increased the number of Nb atoms solidly dissolved into the matrix during the forming process, which promoted dissociated eutectic reactions in the matrix, reduced the number of unfused Nb particles in the second phase, and generated more diffuse Laves phases.

**Figure 14:**
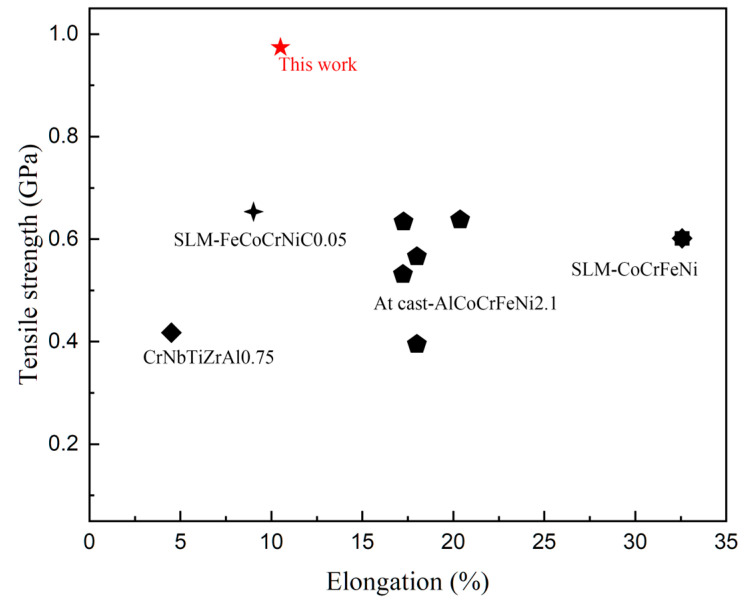
Comparison with other reported HEAs with superior mechanical properties.

Figure 14 [10,20,21,22,23,24,25,26] shows a comparison of the properties of CoCrFeNiNb_0.25_ alloy prepared by mechanical powder mixing and the SLM + LR process with other reported HEA alloys. It was seen that the tensile strength significantly increased after strengthening by the Nb element, but the plasticity significantly reduced compared with the CoCrFeNi matrix alloy; nevertheless, the strengthening effect of the Nb element was still better than that of the C element.

**Figure 15:**
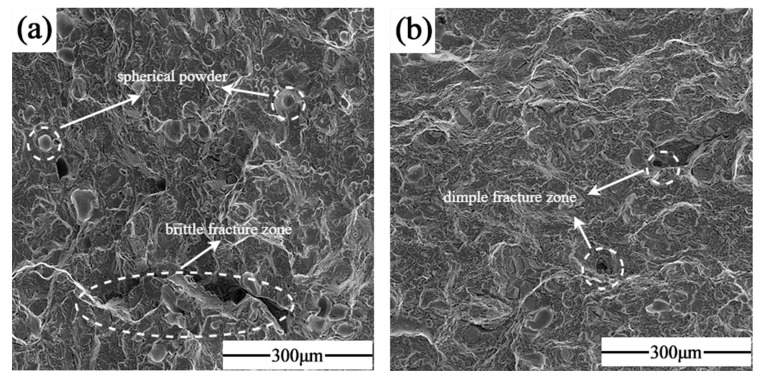
Fracture morphology: (**a**) SLM; (**b**) SLM + LR.

Figure 15 shows the SEM images of the fracture morphology of the samples formed by single scanning forming and laser remelting process, respectively, based on optimized process parameters. (a) Represents the tensile fracture of the specimen that was not subjected to the LR process. Dimples were visible on the fracture surface, and cracks extending along the pores were also observed, which was indicative of a mixed fracture. Additionally, a considerable number of unfused Nb particles were observed in the fracture process, as evidenced by the matrix shedding and the formation of craters. This suggests that the second phase of Nb particles and the matrix exhibited weak mechanical properties, which may have been the primary cause of the fracture failure. (b) Shows the tensile fracture of the specimen formed by using the LR process. It was observed that there was minimal crack extension along the pore defects, accompanied by an increase in the number of fine dimples and a significant decrease in the number of unfused particles, on the one hand, because the laser remelting reduced the content of Nb particles and, on the other hand, the remelting process eliminated the two-phase bonding of the stress concentration due to thermal diffusion. The second phase of the Nb particles expanded with the substrate between the transition layer of the Laves phase so that the particles firmly embedded in the substrate, and the bonding strength with the substrate was enhanced. At the same time, the laser remelting process also increased the Nb content in the matrix, forming more dissociated eutectic diffuse Laves phases in the matrix, which improved the strength.

## 4. Conclusions

1. It is practicable to form a CoCrFeNiNb0.25 alloy using mechanical mixing combined with the SLM process; the specimens can form a solid metallurgical bond with a densification of 99.96%.
2. The interlayer remelting process can eliminate the hole defects in the specimen.
3. The unfused Nb particles in the specimen can be reduced by the layer remelting process.
4. The interlayer remelting process can eliminate the stress around the Nb particles, thus improving the mechanical properties. The microhardness of 376 HV, tensile strength of 974 MPa, elongation of 10.51%, and strong plasticity synergistically improve.

## Data Availability

Data are contained within the article.
